# Neutrophil-to-lymphocyte ratio and platelet-to-lymphocyte ratio as predictors of mortality in acute pulmonary embolism: A systematic review and meta-analysis

**DOI:** 10.12669/pjms.40.6.8802

**Published:** 2024-07

**Authors:** Shanshan Tang, Yanhua Hu

**Affiliations:** 1 Shanshan Tang, Department of Respiratory Medicine, First People’s Hospital of Linping District, 369 Yingbin Road, Hangzhou City, Zhejiang Province 311100, P.R. China; 2 Yanhua Hu, Department of Respiratory Medicine, First People’s Hospital of Linping District, 369 Yingbin Road, Hangzhou City, Zhejiang Province 311100, P.R. China

**Keywords:** Neutrophil-to-lymphocyte ratio, Platelet-to-lymphocyte ratio, Pulmonary embolism, Meta analysis

## Abstract

**Objective:**

The purpose of this review was to examine the association between neutrophil-to-lymphocyte ratio (NLR) and platelet-to-lymphocyte ratio (PLR) and mortality rates in patients with acute pulmonary embolism (PE).

**Methods:**

PubMed Central, Scopus, Web of Science, and Embase were searched for studies reporting the association between NLR and PLR with mortality up to March 17^th^ 2023. Adjusted ratios were sourced from studies and combined to generate pooled outcomes as odds ratio (OR) in a random-effects model. Risk of bias was assessed using the Newcastle Ottawa Scale.

**Results:**

Fifteen studies were included. Meta-analysis showed that NLR was a significant predictor of mortality in patients with PE (OR: 1.42 95% CI: 1.26, 1.61 I^2^=92%). Results were unchanged on sensitivity analysis and subgroup analysis based on study location, method of diagnosis, sample size, overall mortality rates, cut-offs, and follow-up. Pooled analysis failed to demonstrate PLR as a predictor of mortality in patients with PE (OR: 1.00 95% CI: 1.00, 1.01 I^2^=57%). Results were unchanged on sensitivity analysis and subgroup analysis based on study location, diagnosis of PE, overall mortality rates, and cut-off.

**Conclusion:**

Current evidence from retrospective studies shows that NLR can independently predict mortality in acute PE. Data on PLR was limited and failed to indicate an independent role in the prognosis of PE patients.

***Registration No*.** PROSPERO (CRD42023407573).

## INTRODUCTION

Acute pulmonary embolism (PE) is a common thromboembolic disorder seen worldwide.^1^ Clinically, PE that is large enough to cause significant hemodynamic compromise can result in remarkable morbidity and mortality.^2^ Overall mortality rates with PE are around 40% and can reach up to 71.4% for massive cases.^3^ Therefore, risk stratification with mortality assessment has become an important component in the management of PE patients.^4^ For prognostication, the Pulmonary Embolism Severity Index (PESI) and its simplified version (sPESI) are commonly used.^5^ These tools are excellent at identifying low-risk patients. However, for high-risk individuals these indices are suboptimal due to their low specificity, resulting in the need for additional testing like echocardiography and a six-minute walk test which can add to the healthcare costs.^6^

Research has shown that blood indices like neutrophil-to-lymphocyte ratio (NLR) and platelet-to-lymphocyte ratio (PLR) can predict prognosis in patients with cancer, ulcerative colitis, Crohn’s disease, stroke, sepsis, and chronic respiratory obstructive disease.^7^–^12^ Similarly, numerous studies have examined the relationship between NLR and PLR and the outcomes of PE.^13^–^16^ While some studies^15^,^16^ indicate that these indices are predictive of mortality, few suggest otherwise.^13^,^14^ Given the variable results, there is a need for a comprehensive systematic review. Previously, Wang et al.^17^ have attempted a meta-analysis on this subject but with only seven studies and pooled only crude data rather than multivariable adjusted data. In view of such limitations, the present study was conducted to perform an updated and detailed review of the prognostic ability of NLR and PLR for predicting mortality in PE patients.

## METHODS

The systematic review protocol was registered on PROSPERO (CRD42023407573). The PRISMA statement reporting guidelines were followed.^18^ PubMed, CENTRAL, Scopus, Web of Science, and Embase were searched by two reviewers up to 17^th^ March 2023. Only English language publications were considered.

### Inclusion criteria:

- Studies conducted on PE patients.
- Assessing the prognostic ability of NLR or PLR for mortality rates.
- Studies reporting mortality data as multivariate-adjusted ratios. There was no restriction on the sample size, follow-up duration, or cut-off of NLR or PLR.

### Exclusion criteria:

- Studies on general venous thromboembolism and not presenting separate data on PE.
- Studies with duplicate/overlapping data. Review articles and editorials were not considered for inclusion.

The search terms used were “venous thromboembolism”, “pulmonary embolism”, “blood indices”, “cellular indices”, “neutrophil-lymphocyte”, “neutrophil-to-lymphocyte”, “NLR”, “platelet-lymphocyte”, “platelet-to-lymphocyte”, “PLR”, and “mortality” (Supplementary Table-I). Duplicates from the search results were removed and the remaining records were carefully inquired about based on the eligibility criteria by two reviewers separately. This was done first at the title/abstract level and then at the full-text level. Articles completing all eligibility criteria were included. The references list of eligible articles was hand searched for additional articles.

### Data management and study quality:

Data on the author’s last name, year of publication, location, study type, diagnosis of PE, sample size, age, gender, use of thrombolysis, overall mortality rates, the timing of mortality, NLR or PLR cut-off, the method to determine cut-off, and outcome ratios were extracted by two reviewers independent of each other.

Two authors judged the study’s quality based on Newcastle Ottawa Scale (NOS).^19^ The NOS has three domains: representativeness of the study cohort, comparability, and measurement of outcomes.

### Statistical analysis:

Statistical analysis was done using “Review Manager” (RevMan, version 5.3; Nordic Cochrane Centre (Cochrane Collaboration), Copenhagen, Denmark; 2014). Adjusted ratios were sourced from studies and combined to generate pooled outcomes as odds ratio (OR) with 95% confidence intervals (CI) in a random-effects model. Publication bias was examined using funnel plots. The I^2^ statistic was the tool to determine inter-study heterogeneity. I^2^ <50% meant low and >50% meant substantial heterogeneity. A leave-one-out analysis was performed to check for any change in the results on the exclusion of any study. Subgroup analysis was done based on study location, method of diagnosis, sample size, overall mortality rates, cut-offs, and follow-up. P-value <0.05 was considered statistically significant.

## RESULTS

**Fig.1:**
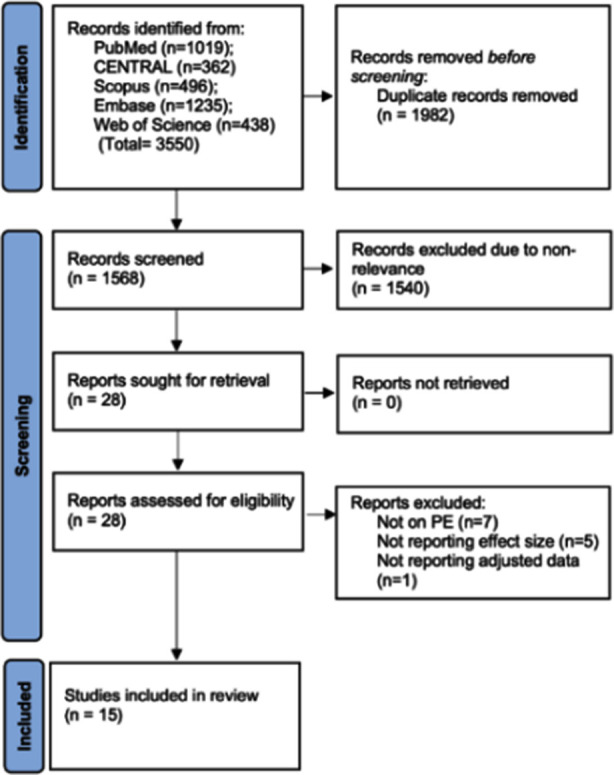
Study flow-chart.

Total 3550 studies were retrieved (Fig.1). Duplicates amongst those were removed leaving 1568 results. One thousand five hundred forty of those were excluded due to non-relevance and 28 studies were selected for full-text analysis and 15^9^,^13^-^16^,^20^-^29^ were included.

The majority of the studies were from Turkey (Table-I). All were retrospective studies. NLR or PLR ratios were measured from the blood samples obtained at admission. The majority of studies used computed tomography pulmonary angiogram (CTPA) for PE diagnosis. Two studies used CTPA or ventilation-perfusion scan while two did not mention the diagnostic criteria for PE. The sample size of the studies varied from 82 to 4487 patients. Thirteen studies reported data on NLR while six studies mentioned PLR. The NLR cut-off ranged from 3.25 to 8.4 while the PLR cut-off varied from 170 to 325. The overall mortality rates in the cohorts ranged from 3.7 to 26.2%. Mortality was calculated either in-hospital, 30-days, 90-days, or long-term (≥1 year). NOS scores of the studies ranged from seven to eight.

**Table-I:** Details of included studies

Study	Location	Diagnosis of PE	Sample size	Mean age	Male gender (%)	Thrombolysis (%)	Mortality (%)	NLR cut-off	PLR cut-off	Cut-off method	Mortality timing	NOS score
Akgullu 2014^20^	Turkey	CTPA	206	61.8	47	12.1	14.5	8.4	-	ROC	IH	7
Cavus 2014^21^	Turkey	CTPA	266	64.8	44.7	NR	6	5.465	-	ROC	30-days	7
Kayrak 2014^9^	Turkey	CTPA	359	63.6	46.8	19.8	14	9.2	-	ROC	30-days	7
Karatas 2016^14^	Turkey	D-dimer, Echo, CTPA	241	65.8	43	21	7	5.93	191	ROC	30-days	7
Ma 2016^23^	China	CTPA	248	66.7	56	0	8	5.99	325	ROC	30-days	7
Soylu 2016^22^	Turkey	Clinical	142	58.9	59.8	28.9	11	5.7	-	ROC	IH	7
Cetin 2017^27^	Turkey	CTPA	459	66	34.9	6.3	17.6	-	149.1	ROC	Long term	8
Telo 2018^25^	Turkey	CTPA	82	61.2	48.7	NR	3.7	-	156	ROC	90-days	8
Sabri 2019^13^	Turkey	CTPA	550	68	50.3	NR	13.8	7.3	170	ROC	30-days	7
Kose 2020^26^	Turkey	NR	103	67.6	44.7	22.3	26.2	NR	NR	NR	Long term	8
Liu 2020^27^	China	CTPA	101	64	40	NR	23.8	NR	-	NR	30-days	7
Duman 2021^28^	Turkey	CTPA	828	62	47	NR	8.5	3.25	-	ROC	Long term	8
Efros 2021^16^	Israel	NR	2072	73	42.5	NR	8.9	5.12	-	Median	30-days	7
Slajus 2021^29^	USA	CTPA or VQ scan	228	63	48.7	NR	21	5.5	-	ROC	NR	7
Siddiqui 2022^15^	USA	CTPA or VQ scan	4487	NR	53	NR	11	4.96	-	ROC	90-days	8

NR, not reported; CTPA, computed tomography pulmonary angiogram; VQ, ventilation perfusion; ROC, receiver operating characteristics; NLR, neutrophil lymphocyte ratio; PLR, platelet lymphocyte ratio; PE, pulmonary embolism; IH, in-hospital.

**Fig.2:**
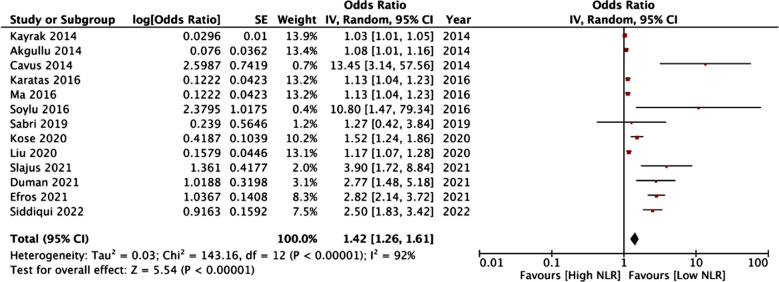
Meta-analysis of the association between NLR and mortality in PE patients.

Meta-analysis showed that NLR was a significant predictor of mortality in patients with PE (OR: 1.42 95% CI: 1.26, 1.61 I^2^=92%) Fig.2. The results remained significant on the sequential exclusion of individual studies. There was some asymmetry on the funnel plot (Supplementary Fig.1). We noted no change in the significance of the results on segregating the studies based on location (Turkish or non-Turkish), diagnostic method of PE (CTPA or others), sample size (>250 or <250), and overall mortality rates in the cohort (>10% or <10%) Table-II. Since most of the studies used an NLR cut-off ranging between four to six, we divided the studies with cut-offs of <4, 4-6, and >6 only to find no change in the significance of the results. Based on the timing of mortality, the results were non-significant for in-hospital mortality but not for other periods.

**Fig.3:**
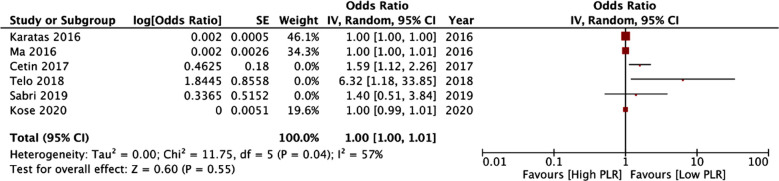
Meta-analysis of the association between PLR and mortality in PE patients.

Pooled analysis showed that PLR was not a statistically significant predictor of mortality in patients with PE (OR: 1.00 95% CI: 1.00, 1.01 I^2^=57%) Fig.3. The results did not change on sensitivity analysis. No publication bias was noted on the funnel plot. Fig.2 There was no change in the significance of the results on subgroup analysis based on location, diagnosis of PE, overall mortality rates, and cut-off. Table-III. However, a meta-analysis of two studies and a singular study indicated that PLR was a significant predictor of mortality in studies with sample size >250 and at follow-up of 90-days respectively.

## DISCUSSION

The widespread prevalence of PE combined with high mortality rates necessitates accurate prognostication of patients to improve survival. Early detection of high-risk patients can help clinicians divert resources and prioritize treatment. Several different markers of mortality have been reported for PE, namely, PESI, sPESI scores, C-reactive protein (CRP), troponin, N-terminal pro–B-type natriuretic peptide (NT-proBNP), Heart-type fatty acid-binding protein, and D-dimer.^30^ The limitations of sPESI for high-risk groups^6^ and the expensive and resource-dependent nature of other markers warrant the development of easy-to-use, inexpensive, widely available, and accurate mortality indicators. In this context, cellular indices offer an interesting domain of risk assessment. Irrespective of the healthcare setup and severity of the disease, all admitted PE patients usually undergo complete blood counts and calculation of indices like NLR and PLR is simple and can be done on bedside. The caveat lies in the accuracy of these markers in predicting mortality.

This meta-analysis showed that high NLR is a significant predictor of mortality in patients with acute PE. However, no relationship was noted between PLR and post-PE mortality. Our results partially conform with the previous meta-analysis of Wang et al.,^17^ wherein they pooled data from seven studies show that patients with elevated NLR had a ten times increased risk of mortality while increased PLR was associated with six times higher risk of death after PE. The significantly higher risk of mortality with both cellular indices in their review could be due to the small number of studies in the meta-analysis and use of crude data. The risk of mortality after PE is confounded by several variables ranging from age, gender, baseline comorbidities, the severity of PE, therapeutic options, etc.^4^ Gauging the risk of mortality based on only crude data overestimates the effect size leading to false conclusions.^31^ The current review not only provides a more robust and updated statistical analysis but also generates a more realistic relationship between the cellular indices and outcomes after PE.

Blood cellular indices are markers of inflammation and research shows that inflammation plays an important role in the pathogenesis of PE. The development of a thrombus is correlated with vascular inflammation and early extravasation of leukocytes. During the acute phase, proinflammatory cytokines and acute-phase proteins like CRP, IL-8, and tumor necrosis factor are released which encourage a procoagulant state by increasing the expression of tissue factors. Also, factors like polyphosphates and bradykinin can trigger the contact systems leading to the activation of external coagulation pathways.^32^,^33^ Also, acute PE is associated with reperfusion injury which increases the oxidative stress and levels of myeloperoxidase enzyme and reactive oxygen species in the lung.^34^

Focusing on specific functions of individual cellular components, neutrophils constitute the first leucocytes to act as the site of PE. They migrate at the site of injury and release proinflammatory mediators and procoagulants that lead to oxidative and proteolytic injury.^32^,^33^ Increased platelet count corresponds to increased thrombocyte activity causing a destructive pro-inflammatory and pro-thrombotic response.^35^ On the contrary lymphocytes counter this reaction by controlling and restraining the increased inflammatory process.^24^ Therefore, a combination of pro-inflammatory cells (neutrophils or platelets) along with a counteracting component (lymphocyte) is an efficient marker of inflammation predicting the prognosis of numerous cardiovascular diseases.^35^ However, pooled analysis of studies in the case of PE showed that only NLR was an independent marker of mortality. No such relationship was noted for PLR. One probable reason could be the limited number of studies available for a meta-analysis of PLR.

The high inter-study heterogeneity in the meta-analysis is a cause of concern. The cause could be primarily due to the varied study populations, differences in baseline health and severity of disease as well as differences in treatment protocols in different setups. Subgroup analysis to overcome the heterogeneity was conducted based on several variables but with little change in the significance of the results. The lack of major change in subgroup analysis and consistency of results on sensitivity analysis add to the credibility of our outcomes.

### Limitations:

The retrospective nature of studies is the biggest drawback due to its inherent selection bias. Secondly, significant variations in cut-offs of NLR and PLR were noted in the review. Studies calculated separate cut-offs in their cohorts to establish the prognostic ability of cellular indices. To optimize their use globally, there needs to be a multicentric worldwide study to generate optimal cut-offs of these easy-to-use indices. Thirdly, while the number of studies in the meta-analysis of NLR was high the same was not true for PLR. Lastly, the majority of the studies were from a single country which greatly limits the generalizability of evidence.

## CONCLUSIONS

Current evidence from retrospective studies shows that NLR can independently predict mortality in acute PE. Data on PLR was limited and failed to indicate an independent role in the prognosis of PE patients. Further studies from across the world are needed to increase the credibility of the evidence.

### Authors’ contributions:

**ST** conceived and designed the study.

**ST and YH** collected the data and performed the analysis.

**ST** was involved in the writing of the manuscript and is responsible for the integrity of the study.

All authors have read and approved the final manuscript
